# Latitudinal Variation in the Phenology of the Emergent Species *Phragmites australis* and the Submerged Species *Potamogeton crispus* in Shallow Lakes of the North China Plain

**DOI:** 10.3390/plants15203240

**Published:** 2026-10-21

**Authors:** Jia Tang, Quanzhou Yu, Yong Li, Hao Zhang, Jun Ma, Shuwen Xiao, Tianquan Liang, Qingxin Tang, Shaoqiang Wang

**Affiliations:** 1 School of Geography and Environment, Liaocheng University, Liaocheng 252059, China; 2 Key Laboratory of Ecosystem Network Observation and Modeling, Institute of Geographic Sciences and Natural Resources Research, Chinese Academy of Sciences, Beijing 100101, China; 3 School of Land Resources and Surveying and Mapping Engineering, Shandong Agriculture and Engineering University, Jinan 250100, China; 4 Shandong Huaao Geographic Information Co., Ltd., Jinan 250000, China

**Keywords:** shallow lakes, aquatic plant phenology, latitudinal zonality, environmental associations, North China Plain

## Abstract

Phenology, as a pivotal functional trait, serves as an important indicator for understanding vegetation responses to environmental variability. Against the backdrop of climate change, the latitudinal zonation of vegetation phenology has attracted widespread attention. However, how the phenology of different aquatic plants responds to latitudinal variation remains insufficiently understood. To characterize the contrasting responses of aquatic vegetation (submerged and emergent) along a latitudinal gradient, this study focuses on the submerged species *Potamogeton crispus* (*P. crispus*) and the emergent species *Phragmites australis* (*P. australis*), both of which are widely distributed across shallow lakes on the North China Plain. Based on dense time-series Normalized Difference Vegetation Index (NDVI) data from Sentinel-2 and a standardized phenological extraction method, we analyzed the spatial differentiation patterns of these two vegetation types, as well as their latitudinal phenological responses and environmental associations, including the Start of the growing Season (SOS), End of the growing Season (EOS), and length of the growing season (LOS). The results showed marked differences in the spatial distributions of *P. crispus* and *P. australis*, reflecting their distinct ecological niches within lake ecosystems. Their phenological metrics also exhibited contrasting latitudinal patterns. For *P. australis*, SOS showed no significant latitudinal trend, whereas EOS occurred progressively earlier and LOS became shorter with increasing latitude. For *P. crispus*, both SOS and EOS were delayed with increasing latitude, while LOS showed no significant latitudinal gradient. Among the linear predictors, annual mean temperature showed the highest overall importance, but its associations with the phenology of *P. australis* and *P. crispus* differed markedly. Other environmental and anthropogenic factors also showed species- and stage-specific associations with phenological variation. XGBoost-SHAP analysis (Extreme Gradient Boosting (XGBoost) combined with SHapley Additive exPlanations (SHAP)) further revealed nonlinear marginal associations between selected environmental predictors and phenological metrics, including an apparent change in the modeled EOS response of *P. australis* near 16 °C, which should be interpreted as an exploratory model-derived feature rather than a confirmed threshold. This study highlights contrasting latitudinal phenological responses of submerged and emergent macrophytes and provides scientific support for ecological monitoring and management of shallow lake ecosystems in the North China Plain.

## 1. Introduction

Vegetation phenology is a key ecological indicator of seasonal vegetation dynamics and ecosystem responses to environmental change. It provides important information on seasonal ecosystem dynamics and is widely used to assess ecological responses to environmental variability and global change [1]. Changes in vegetation phenology can influence ecosystem primary productivity, carbon balance, energy exchange, and hydrological processes, thereby affecting ecosystem structure and functioning [2]. Under the combined influence of global climate change and anthropogenic activities, vegetation phenology exhibits pronounced spatial variation and systematic patterns along environmental gradients.

Latitudinal variation in vegetation phenology has been widely documented across large spatial gradients [3]. Large-scale latitudinal gradients in thermal conditions drive systematic spatial variation in vegetation phenology [4]. Moreover, vegetation functional types differ markedly in their sensitivity to north–south thermal gradients [2,5], and such contrasting responses are linked to differences in climatic sensitivity and plant functional traits [6,7]. Elevational gradients also generate vertical variation in vegetation phenology through associated changes in temperature and water availability [8,9], and urban–rural gradients associated with human activities have been linked to differences in vegetation Start of the growing Season (SOS) and End of the growing Season (EOS) [10]. Overall, research on terrestrial vegetation phenology has yielded a relatively well-established understanding of spatial patterns and environmental responses across forests, grasslands, and croplands. However, whether similar patterns occur in aquatic vegetation remains insufficiently understood.

Compared with terrestrial vegetation, phenological research on aquatic vegetation has received considerably less attention and therefore warrants further investigation. Aquatic vegetation occupies diverse habitats spanning submerged, floating, and emergent environments in lakes and wetlands and provides important ecological functions, including water-quality regulation, habitat provision, biodiversity maintenance, and carbon sequestration [11,12]. Because aquatic vegetation is closely coupled with both terrestrial and aquatic environments, its phenology can be influenced by multiple factors, including climate, hydrology, water quality, and human activities, potentially resulting in phenological responses that differ from those of terrestrial vegetation [13]. Long-term remote sensing studies have demonstrated the feasibility of monitoring aquatic vegetation phenology in individual lakes. However, most existing studies have remained geographically localized, focusing on a single lake or species [14,15]. For example, Ojdanič et al. investigated *Phragmites australis* (*P. australis*) in Lake Čerknica, Slovenia, and used time-series Normalized Difference Vegetation Index (NDVI) to identify intra-annual phenological stages and examine the association between local hydrological conditions and the phenology of *P. australis* [16]. Liu et al. used time-series Sentinel imagery to identify the submerged species *Potamogeton crispus* (*P. crispus*) in Baoan Lake, Baiyang Lake, Ulansu Lake, and Dongping Lake, but did not examine in depth the environmental factors associated with its phenological variation [17]. Yang et al. conducted a long-term time-series analysis of *P. australis* in Gaoyou Lake and examined the relationships between local hydrometeorological conditions and its distribution dynamics [18]. Collectively, these studies demonstrate the potential of remote sensing for monitoring aquatic vegetation phenology, but their predominantly local spatial coverage limits our understanding of phenological variation across broader environmental gradients.

Several limitations remain in large-scale studies of aquatic vegetation phenology. Existing studies largely rely on medium- or low-resolution remote sensing data, such as MODIS and Landsat. Their relatively coarse spatial resolution makes it difficult to reliably identify fragmented patches of emergent and submerged vegetation in shallow lakes, thereby limiting the characterization of fine-scale phenological differences among vegetation types [19,20]. The relatively fine spatial and temporal resolution of Sentinel-2 provides an opportunity to improve species-level monitoring of fragmented aquatic vegetation and to construct denser NDVI time series for phenological retrieval. In addition, relatively few studies have examined aquatic vegetation phenology across broad latitudinal gradients, where large-scale climatic and thermal conditions may generate systematic spatial variation [21]. More importantly, emergent and submerged vegetation are often considered collectively, and comparatively few studies have explicitly examined whether these functionally distinct vegetation types exhibit different latitudinal phenological responses [22]. Emergent and submerged plants differ substantially in their growth environments and physiological characteristics. Consequently, their sensitivities to latitudinal thermal gradients and the environmental factors associated with their phenological responses may also differ. However, such functional-type-specific responses remain insufficiently understood, representing an important gap in large-scale aquatic vegetation phenology research. In particular, it remains unclear whether contrasting aquatic plant functional types exhibit consistent or divergent phenological responses along the same latitudinal gradient, and which environmental factors are associated with these differences.

To address these gaps, this study investigates five shallow lakes spanning approximately 3.5° of latitude along the East Route of the South-to-North Water Diversion Project (ESNWD) on the North China Plain, focusing on the typical emergent species *P. australis* and the submerged species *P. crispus*. Rather than focusing on a single lake or species, we established a multi-lake comparative framework to examine whether aquatic plants with contrasting growth forms exhibit divergent phenological responses along a common latitudinal gradient. Using dense Sentinel-2 time series, species-specific vegetation mapping and a consistent phenological extraction framework based on Double Logistic (DL) reconstruction, we derived SOS, EOS, and Length of the growing Season (LOS) for both species and quantified their spatial and latitudinal patterns. We further combined correlation and regression analyses with Partial Least Squares Regression (PLSR) and Extreme Gradient Boosting (XGBoost) coupled with SHapley Additive exPlanations (SHAP) (XGBoost-SHAP) to evaluate both linear associations and exploratory nonlinear relationships between phenology and climatic, hydrological, aquatic environmental, and anthropogenic factors. This integrated framework enables us to move beyond the description of local phenological patterns and provide a comparative assessment of how different aquatic plant functional types respond to the same latitudinal environmental gradient. Because water-temperature observations were unavailable, air temperature was used as a climatic indicator, and the resulting associations should not be interpreted as direct evidence of aquatic thermal mechanisms. The primary contribution of this study is therefore not the development of a new remote-sensing or statistical algorithm, but the integration of fine-resolution satellite time series and complementary analytical approaches to reveal species-specific latitudinal phenological patterns across multiple shallow lakes.

Through this design, we extend species-level aquatic vegetation phenology monitoring from single-lake investigations to a regional multi-lake latitudinal comparison. By leveraging Sentinel-2 fine-resolution time series for fragmented lake vegetation and combining linear and exploratory nonlinear modeling to link phenological variation with environmental drivers, this work delivers new comparative evidence for divergent latitudinal responses between emergent and submerged macrophytes.

The main objectives of this study are: (1) to characterize the spatial distribution patterns of *P. australis* and *P. crispus* across different lake habitats and phenological stages; (2) to compare the latitudinal responses of the phenological parameters of these two aquatic vegetation types; and (3) to identify the climatic, hydrological, aquatic environmental, and anthropogenic factors associated with their phenological variation and characterize their linear and nonlinear responses to multiple environmental factors.

## 2. Materials and Methods

### 2.1. Study Area

The study area comprises a group of lakes located along the water conveyance channel of the ESNWD on the North China Plain, including Dongping Lake (DPL), the Nansi Lake North (NSLN), the Nansi Lake South (NSLS), Luoma Lake (LML), and Gaoyou Lake (GYL) [23]. Geographically, the study area extends from approximately 32°30′ to 36°00′ N and 116°30′ to 119°30′ E, spanning the transition zone between the warm-temperate and northern subtropical climatic zones and exhibiting a pronounced latitudinal climatic gradient. These five shallow lowland lakes lie on the North China Plain, where terrestrial elevation varies only slightly across sampling sites. Such limited elevation differences exert negligible impacts on aquatic vegetation phenology, making latitudinal climate the primary gradient examined rather than altitude. The lakes share broadly similar ecological functions but differ systematically along the regional climatic gradient. Collectively, they represent typical shallow freshwater lakes in eastern China, featuring gentle lake basins and shallow water. Lake surface water level (5–42 m) was incorporated as an environmental predictor in subsequent driver analyses. The lakes have diverse water sources and support aquatic vegetation dominated by *P. australis* and *P. crispus* [24]. Hongze Lake is shown in the map for geographic context but was not included in the analysis, because of insufficient imagery quality and large temporal gaps between mosaicked scenes that would affect phenological extraction.

The lakes are distributed from north to south across the densely populated provinces of Shandong and Jiangsu, spanning approximately 3.5° of latitude. Human activities are widespread throughout the region [25]. These lakes are connected by the ESNWD conveyance channel and share similar lake type and hydrological background, which minimizes confounding environmental heterogeneity and thereby provides favorable conditions for isolating the latitudinal effect on aquatic vegetation phenology. The pronounced seasonal monsoon climate produces distinct seasonal variations in temperature and hydrological conditions, providing a suitable natural setting for comparative analysis of aquatic vegetation phenology along a latitudinal gradient. This ~3.5° latitudinal transect captures the warm-temperate to northern subtropical transition and enables analysis of regional latitudinal patterns; however, as it is limited to eastern lowland shallow lakes, extrapolation to the national scale should be treated cautiously.

### 2.2. Data and Preprocessing

The datasets used in this study are summarized in Table 1. Sentinel-2 Level-2A (L2A) imagery from 2022 to 2024 served as the primary remote sensing data source. The imagery provides a spatial resolution of 10–60 m and a nominal revisit interval of approximately 5 days, enabling relatively fine-scale monitoring of aquatic vegetation dynamics. Temporal gaps caused by cloud contamination were supplemented using observations from adjacent image acquisitions. The preprocessing workflow included cloud and cloud-shadow masking, clipping to lake boundaries, and construction of NDVI time series. The NDVI time series were subsequently fitted and reconstructed using the DL function in TIMESAT 3.3 (Lund University, Lund, Sweden). In addition, auxiliary datasets were incorporated to characterize climatic, hydrological, water-quality, and anthropogenic conditions potentially associated with aquatic vegetation phenology.

### 2.3. Methods

#### 2.3.1. Vegetation Classification and Index Calculation

*P. australis* was extracted using the Random Forest (RF) algorithm based on Sentinel-2 L2A imagery acquired during its peak growth period (Table 2). The predictor variables included Sentinel-2 spectral bands and derived remote sensing indices, including NDVI, Enhanced Vegetation Index (EVI), Normalized Difference Water Index (NDWI), and Normalized Difference Aquatic Vegetation Index (NDAVI) (Table 3). These spectral and spectral-index features were used to enhance the separability of *P. australis* from other land-cover classes. Classification accuracy was evaluated using a confusion matrix, with overall accuracy (OA) and the Kappa coefficient used to assess classification performance.

*P. crispus* was extracted using a temporal NDVI feature-based method [26,27]. Sentinel-2 NDVI images corresponding to the vigorous-growth and senescence periods of *P. crispus* were selected (Table 2). First, non-target land-cover types, such as cropland, were excluded using an NDVI threshold of >0.5. The inter-date NDVI difference was then calculated, pixels with an NDVI difference > 0.1 were identified as areas of *P. crispus* distribution [26]. Producer accuracy (PA) was subsequently used to evaluate the extraction results.

Reference samples were generated via visual interpretation of Sentinel-2 imagery. For each lake and each year, approximately 50 annotated reference sites were selected for training the *P. australis* RF classification, and a separate set of 30 annotated reference sites was selected for independent validation of *P. australis*. Validation pixels for *P. australis* were spatially independent from training pixels within the same lake and year to avoid spatial autocorrelation bias. For *P. crispus*, the temporal difference-based extraction requires no training samples. Its producer’s accuracy was evaluated using a dedicated set of 30 visually interpreted validation sites per lake and year. To further assess commission error, user’s accuracy of *P. crispus* was quantified using an additional ~200 independent random pixels sampled within the mapped *P. crispus* patches. The user’s accuracy of *P. crispus*, together with producer’s and user’s accuracy of *P. australis*, are provided in Table S1 in Supplementary Materials.

#### 2.3.2. Extraction of Phenological Parameters

The DL function implemented in TIMESAT 3.3 was used to fit and reconstruct the Sentinel-2 NDVI time series of *P. australis* and *P. crispus* from 2022 to 2024. The DL function was used to characterize intra-annual vegetation dynamics and reduce the influence of noise associated with cloud contamination and atmospheric effects [28]. The calculation formula is shown below:

(1) $$\mathrm{NDVI}(t)=w+(m-w)\left[\frac{1}{1+e^{-m_{S}(t-S)}}+\frac{1}{1+e^{m_{A}(t-A)}}-1\right]$$

In the equation: *t* denotes time in days; *w* denotes the winter background NDVI; *m* denotes the maximum NDVI during the growing season; *S* and *A* denote the spring and autumn inflection points, respectively; and *m*_S_ and *m*_A_ denote the rates of increase and decrease, respectively. After fitting, the reconstructed NDVI curves were used to extract phenological metrics using a dynamic threshold method.

Species-specific threshold values were selected based on the characteristics of the NDVI temporal profiles and previous studies. For *P. australis*, the SOS and EOS thresholds were both set to 0.2, following previous studies on NDVI-based remote sensing monitoring of wetland *P. australis* phenology [16]. As an early-spring submerged macrophyte, *P. crispus* is strongly affected by water-background effects, which can weaken its NDVI signal during the early growth stage. Its NDVI trajectory also exhibits relatively rapid senescence near the end of the growing season. Accordingly, the thresholds for SOS and EOS were set to 0.3 and 0.5, respectively [29]. The fitted DL curves show gradual rising and falling segments. Minor threshold adjustments within these transition ranges only slightly shift the absolute SOS and EOS dates, while the relative latitudinal patterns of phenology remain preserved. For each valid pixel and year, the LOS was calculated as the difference between EOS and SOS (LOS = EOS − SOS). The resulting pixel-level SOS, EOS, and LOS estimates were then spatially aggregated to construct the annual lake-level phenological dataset for the two aquatic vegetation types. Pixels with unrealistic or invalid phenological outputs were excluded prior to aggregation. To reduce the impacts of extreme outliers, the top 5% and bottom 5% quantiles of phenological values from valid pixels were further removed. Lake-level phenological values were calculated as the arithmetic mean of the remaining valid pixels within each lake boundary.

#### 2.3.3. Latitudinal Patterns and Environmental Associations of Phenology

(1) Latitudinal patterns of phenological metrics

Univariate linear regression was used to quantify latitudinal associations in phenological metrics. The latitude of the geometric center of each lake was used as the independent variable, while SOS, EOS, and LOS of *P. australis* and *P. crispus* were separately used as dependent variables. Regression slopes, coefficients of determination (R^2^), and *p* values were used to characterize the direction, strength, and statistical significance of the latitudinal associations.

(2) Environmental associations of phenological metrics

A framework integrating Pearson correlation analysis, PLSR, and XGBoost-SHAP analysis was used to characterize the associations between aquatic vegetation phenology and climatic, hydrological, water-quality, and anthropogenic factors across the lake cluster.

First, Pearson correlation analysis was conducted to screen for linear associations between environmental predictors and phenological metrics, with *p* < 0.05 considered statistically significant. Given our limited sample size (*n* = 15 lake-year observations) and multiple pairwise tests across numerous predictor-metric combinations, we report nominal *p* values without False Discovery Rate (FDR) correction. These correlation results are interpreted as exploratory screening of statistical associations rather than definitive causal evidence. To reduce redundancy among highly correlated predictors, candidate environmental variables were grouped into three categories: meteorological, hydrological and water-quality, and anthropogenic factors. Representative variables were retained from each category for subsequent PLSR and XGBoost-SHAP analyses. The Pearson correlation coefficient was calculated as follows:

(2) $$r=\frac{\sum_{i=1}^{n}(x_{i}-\bar{x})(y_{i}-\bar{y})}{\sqrt{\sum_{i=1}^{n}{\left(x_{i}-\bar{x}\right)}^{2}}\sqrt{\sum_{i=1}^{n}{\left(y_{i}-\bar{y}\right)}^{2}}}$$

In the equation, *r* represents the Pearson correlation coefficient; *x_i_* denotes the value of the independent variable for the i-th sample; *y_i_* denotes the value of the dependent variable for the i-th sample; $\bar{x}$ denotes the mean value of the independent variable; $\bar{y}$ denotes the mean value of the dependent variable; and n denotes the number of samples.

Second, PLSR was used to assess the direction and relative importance of the retained environmental predictors [30]. The optimal number of latent PLS components for each PLSR model was determined via leave-one-out cross-validation (LOOCV) by minimizing the Root Mean PRESS. The cumulative explained variances for predictors (*R*^2^X_cum_) and phenological response variables (*R*^2^Y_cum_) of each PLSR model are summarized in Table S3. Standardized regression coefficients and variable importance in projection (VIP) values were used to characterize the direction and relative importance of predictors, respectively. Predictors with VIP > 1 were considered relatively important. A total of 15 lake-year observations from five lakes during 2022–2024 were included, and the results were interpreted as exploratory associations given the limited sample size.

Finally, XGBoost-SHAP was used to explore potential nonlinear marginal associations between aquatic vegetation phenology and environmental predictors [31]. The 15 lake-year observations were used to model SOS, EOS, and LOS separately. Leave-one-lake-out cross-validation (LOLO-CV) was used to assess cross-lake generalizability, with four lakes (12 observations) for training and one lake (3 observations) for validation in each iteration. Model performance was evaluated using pooled out-of-fold coefficient of determination (*R*^2^_cv_), root mean square error (RMSE), and mean absolute error (MAE).

The XGBoost model used 60 trees, a learning rate of 0.05, a maximum depth of 3, a minimum child weight of 3, subsample and column-subsample ratios of 0.8, and an L2 regularization parameter of 5. The prediction function was expressed as:

(3) $${\overset{\Lambda }{y}}_{i}=\sum_{k=1}^{K}f_{k}(x_{i}),\;f_{k}\in F$$

In the equation, *ŷi* represents the predicted value of the phenological index; *K* denotes the total number of decision trees; *f_k_*(*x_i_*) represents the prediction output of a single decision tree; and *F* denotes the set of all decision trees.

After cross-validation, the XGBoost model was refitted using all 15 lake-year observations. SHAP values were then calculated using the final fitted model to quantify the model-derived marginal contribution of individual predictors to phenological predictions. SHAP dependence plots were used to characterize potential nonlinear response patterns across the observed predictor ranges [32]. The SHAP decomposition can be expressed as:

(4) $$f(x)=\varphi_{0}+\sum_{i=1}^{n}\varphi_{i}$$

where *f*(*x*) denotes the model prediction, *φ*_0_ denotes the expected model output (baseline value), and *φ_i_* denotes the single-factor SHAP value. Positive and negative SHAP values indicate positive and negative contributions to the model prediction, respectively, with larger absolute SHAP values indicating greater contributions.

Given the limited sample size and the cross-lake predictive performance of the XGBoost model, the XGBoost-SHAP results were interpreted as exploratory nonlinear associations rather than causal relationships or robust predictive relationships.

#### 2.3.4. Analytical Workflow

**Figure 1:**
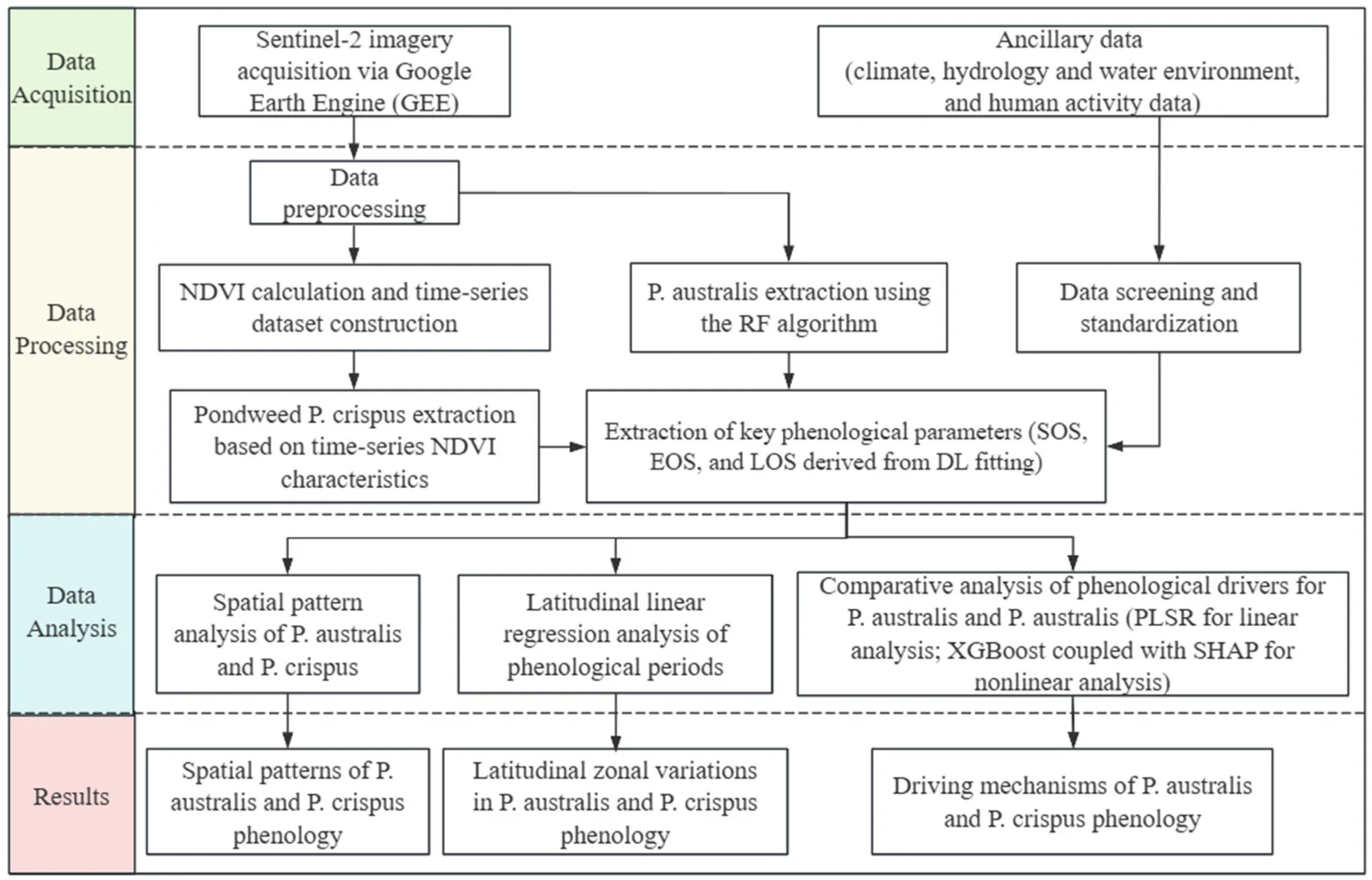
Technical workflow.

This study focused on *P. australis* and *P. crispus* across multiple shallow lakes on the North China Plain. Sentinel-2 time-series imagery was used to construct NDVI time series and map the spatial distributions of the two vegetation types. The NDVI time series of both vegetation types were fitted and reconstructed using the DL function, followed by the extraction of SOS, EOS, and LOS. Spatial patterns and latitudinal trends in phenology were subsequently characterized, followed by the identification of relatively important environmental predictors using PLSR and the exploration of nonlinear associations using XGBoost-SHAP. The overall analytical workflow is presented in Figure 1.

## 3. Results

### 3.1. Distribution Patterns of Typical Vegetation in the Lake Cluster

**Figure 2:**
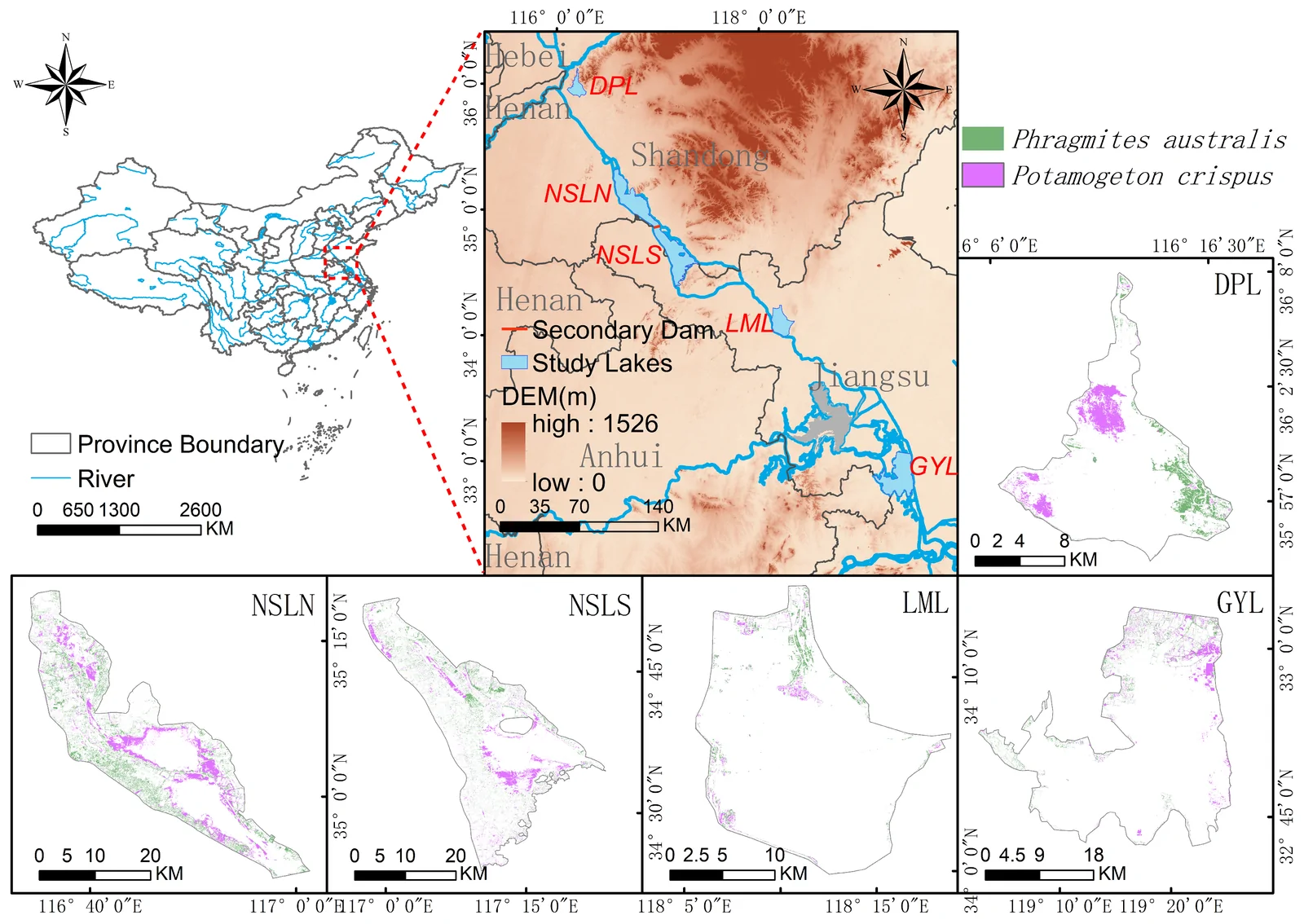
Spatial patterns of *P. australis* and *P. crispus* in the five study lakes (2022–2024). The inset map in the upper left indicates the location of the study area in China. The enlarged subplots show the distribution of *P. australis* (green) and *P. crispus* (purple) within each lake.

Figure 2 shows the spatial distributions of *P. australis* and *P. crispus* across the five study lakes from 2022 to 2024. The two vegetation types exhibited distinct spatial patterns: *P. australis* occurred predominantly in nearshore shallow-water areas and around river inflows, whereas *P. crispus* was mainly distributed in littoral and open-water areas. Overall, the northern lakes had greater areal coverage by the two target macrophytes than the southern lakes, with NSLN showing the highest three-year mean coverage and LML the lowest. *P. crispus* generally occupied a larger proportion of the lake area than *P. australis* in the northern lakes, whereas the opposite pattern was observed in the southern lakes.

The two vegetation types also showed contrasting interannual dynamics (Table 4). Across the five lakes, the total area of *P. crispus* declined from 136.65 km^2^ in 2022 to 100.83 km^2^ in 2023 and 71.86 km^2^ in 2024. Its temporal trajectory varied among lakes, with a pronounced decline in DPL, a decrease followed by partial recovery in NSLN, an increase followed by a marked decline in NSLS, a modest recovery in LML, and a substantial decline in GYL. In contrast, *P. australis* showed more heterogeneous lake-specific changes. Its area decreased from 2022 to 2023 but increased in 2024 in DPL and NSLN, increased markedly in NSLS in 2024, remained relatively stable in LML, and increased progressively in GYL from 11.70 to 16.49 km^2^.

The three-year mean areal proportions further revealed a clear north–south difference in the relative distributions of the two vegetation types. In the northern lakes, *P. crispus* had a higher mean proportion than *P. australis* in DPL (4.03% vs. 3.55%), NSLN (7.36% vs. 5.45%), and NSLS (5.40% vs. 3.24%). In the southern lakes, *P. australis* had a higher mean proportion than *P. crispus* in LML (1.37% vs. 0.70%) and GYL (1.95% vs. 1.86%). Thus, the relative distribution of the two target vegetation types shifted from greater *P. crispus* representation in the northern lakes toward greater *P. australis* representation in the southern lakes.

The accuracy assessment results are summarized in Table 5. The overall accuracy (OA) of the RF classifications for *P. australis* ranged from 94.31% to 99.92%, with Kappa coefficients of 0.80–0.99, while the producer accuracy (PA) for *P. crispus* ranged from 84.91% to 98.70%. These results indicate generally good agreement between the mapped vegetation distributions and the reference samples, supporting their use in subsequent spatial and phenological analyses.

### 3.2. Spatial Patterns of Phenological Metrics of Typical Vegetation in the Lake Cluster

#### 3.2.1. Spatial Patterns of Phenological Metrics of *P. australis*

**Figure 3:**
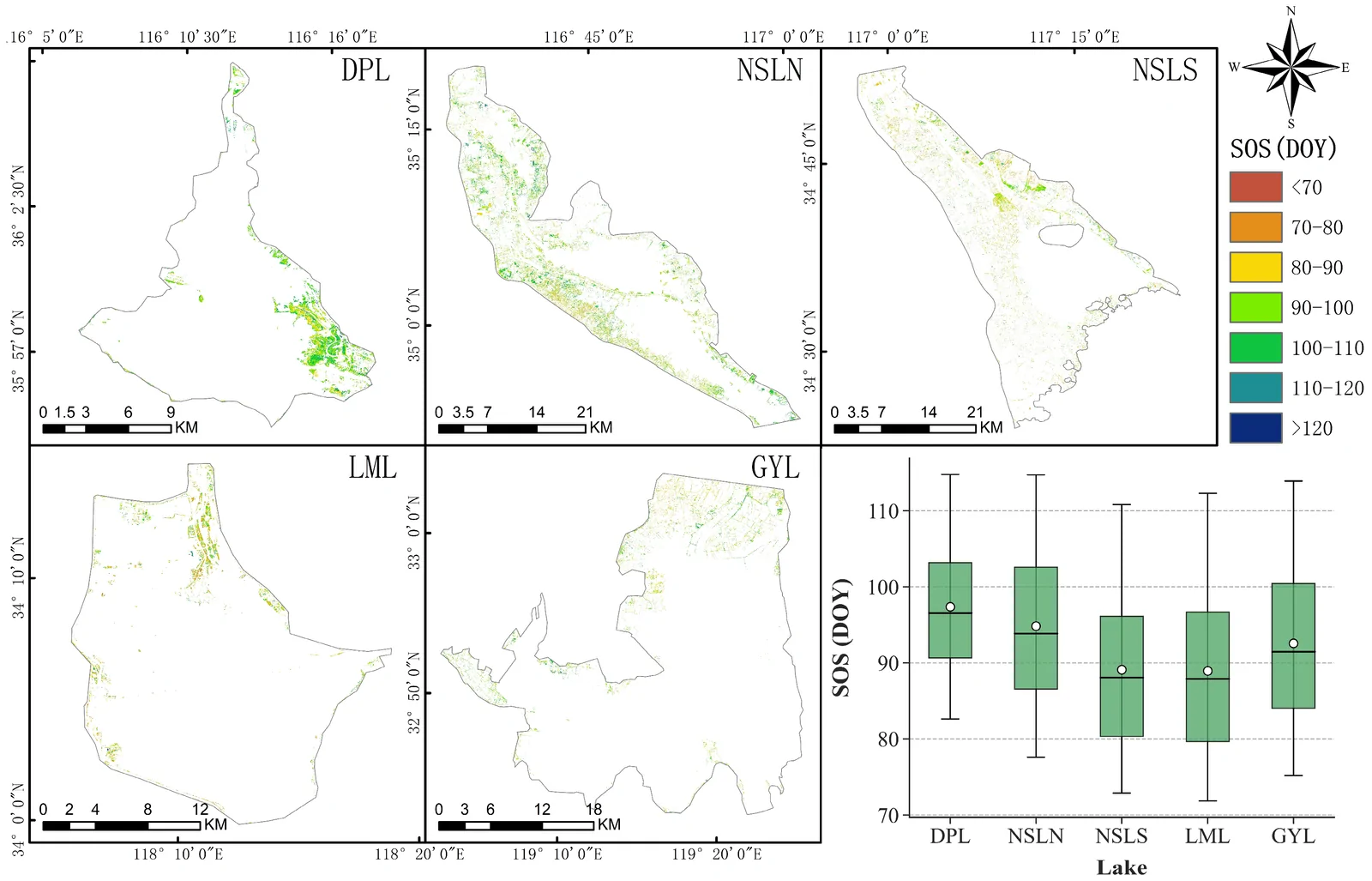
Spatial patterns of SOS for *P. australis* in various lakes.

Overall, the SOS of *P. australis* varied relatively little among the five lakes and showed no significant latitudinal trend (Figure 3). The difference between the highest and lowest lake-level mean SOS values was 8 days. The latest mean SOS occurred in DPL, the northernmost lake, at day of year (DOY) 97, whereas the earliest occurred in LML at DOY 89. GYL, despite being the southernmost lake, showed an intermediate mean SOS. Thus, the spatial pattern of SOS was not strictly ordered along the latitudinal gradient. The relatively earlier SOS observed at lower latitudes may be associated with the regional thermal gradient; however, the absence of a significant latitudinal trend suggests that latitudinal position alone did not explain the observed variation in SOS. The within-lake spatial variability in SOS was also substantial. The standard deviation of SOS within individual lakes averaged approximately 11 days, with the greatest dispersion in LML and the lowest in DPL. This variability indicates considerable spatial heterogeneity in the remotely sensed SOS estimates of *P. australis* across the lake cluster.

**Figure 4:**
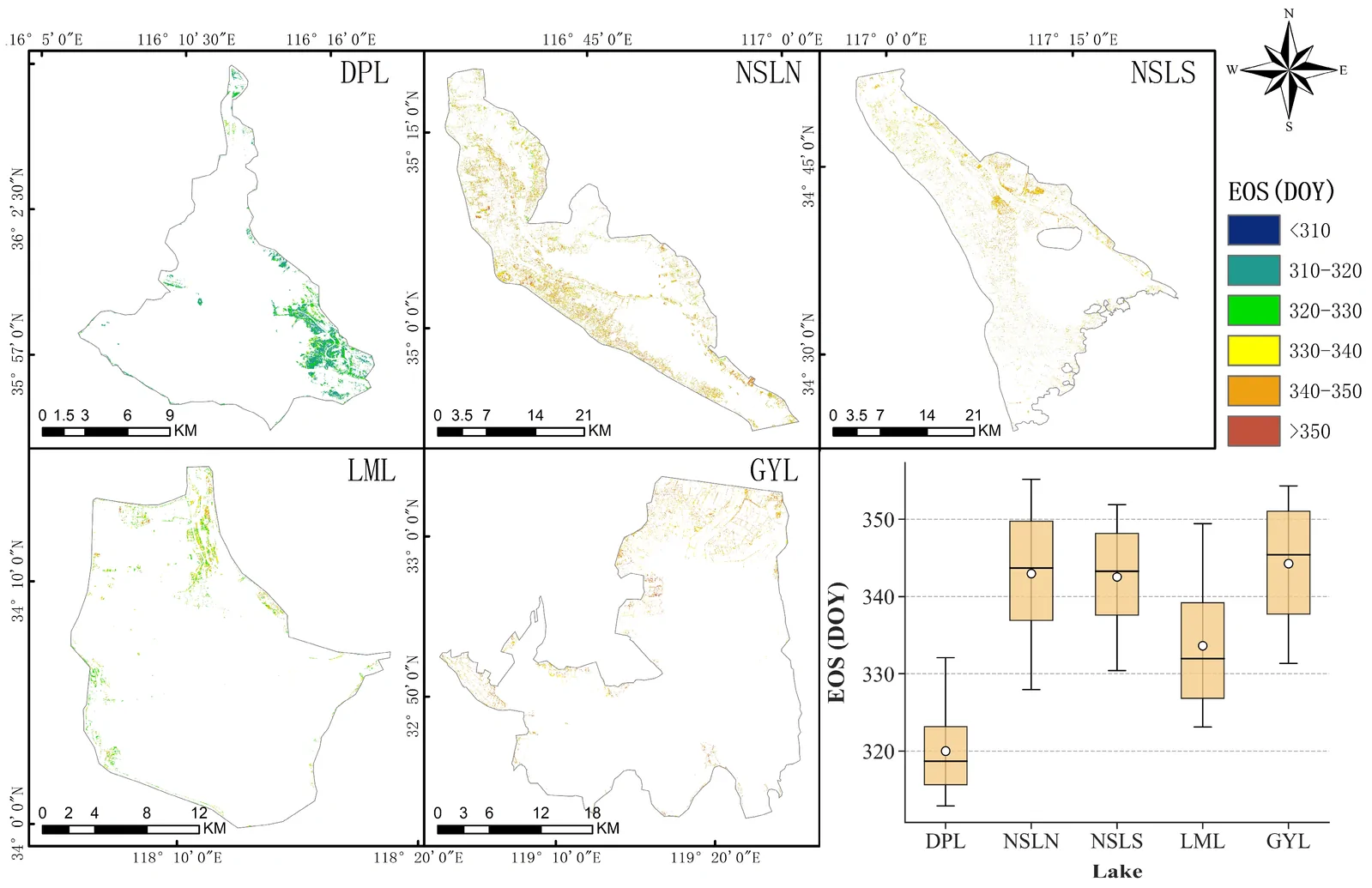
Spatial patterns of EOS for *P. australis* in various lakes.

As shown in Figure 4, the EOS of *P. australis* varied substantially among the lakes and generally occurred earlier with increasing latitude. DPL, the northernmost lake, had the earliest mean EOS at DOY 320, followed by LML at DOY 334, whereas the mean EOS values in the other lakes occurred after DOY 340. The latest mean EOS occurred in GYL, the southernmost lake, at DOY 344, which was 24 days later than that in DPL. The standard deviation of EOS within individual lakes averaged approximately 7 days across the five lakes, which was lower than that of SOS. This result indicates lower within-lake spatial variability and greater spatial consistency in the EOS estimates of *P. australis* than in the corresponding SOS estimates.

**Figure 5:**
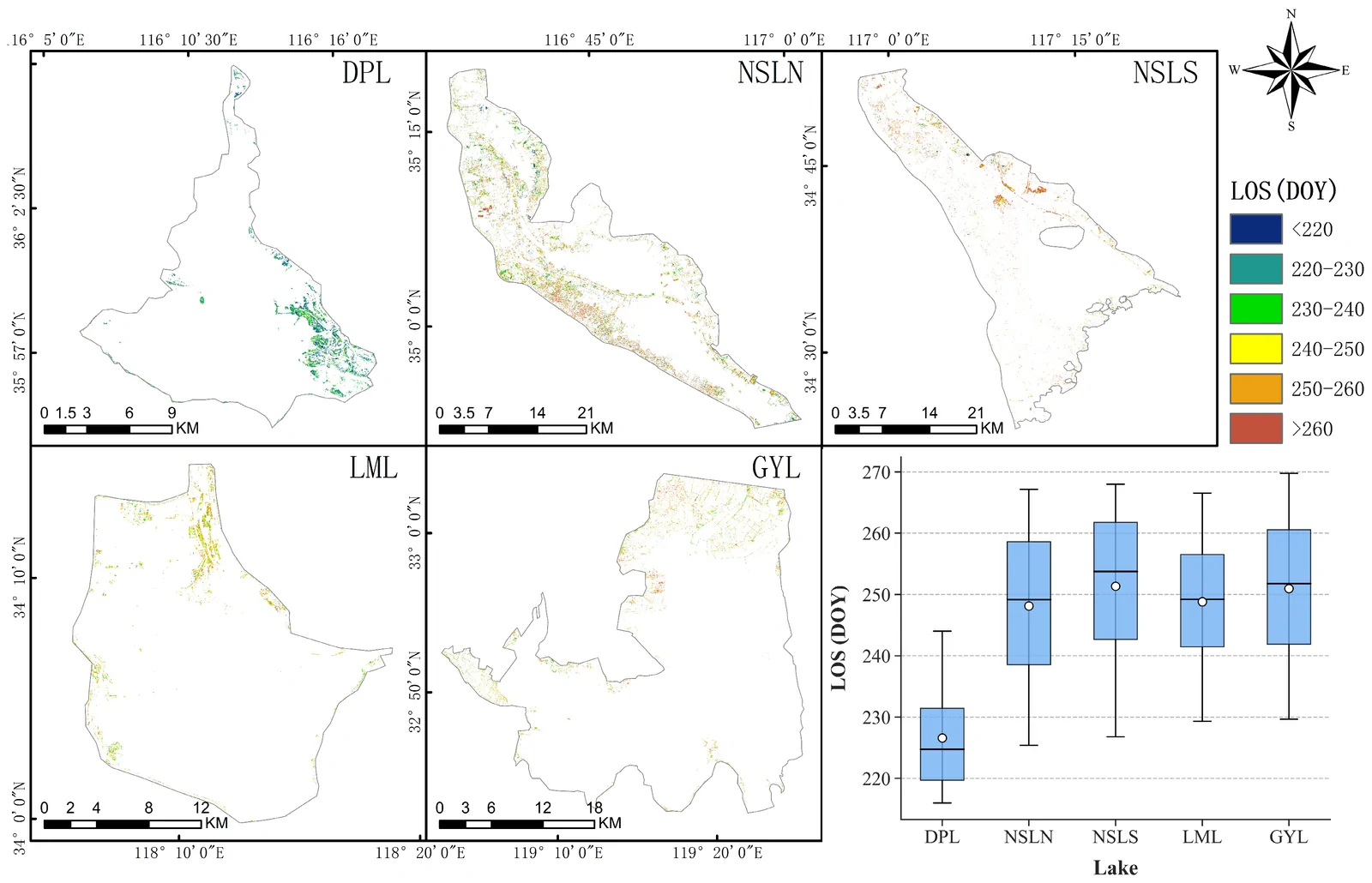
Spatial patterns of LOS for *P. australis* in various lakes.

As shown in Figure 5, the LOS of *P. australis* exhibited a clear latitudinal pattern, with shorter growing seasons in the northern lakes and generally longer growing seasons in the southern lakes. At the lake scale, DPL had the shortest mean LOS, at 227 days, whereas the mean LOS generally increased southward. NSLS, despite being located at an intermediate latitude, had the longest mean LOS, at 254 days. The spatial standard deviation of LOS within individual lakes was relatively large, averaging approximately 11 days across the five lakes, with the greatest variability observed in NSLN and the lowest in DPL.

#### 3.2.2. Spatial Distribution Patterns of Phenological Metrics of *P. crispus*

**Figure 6:**
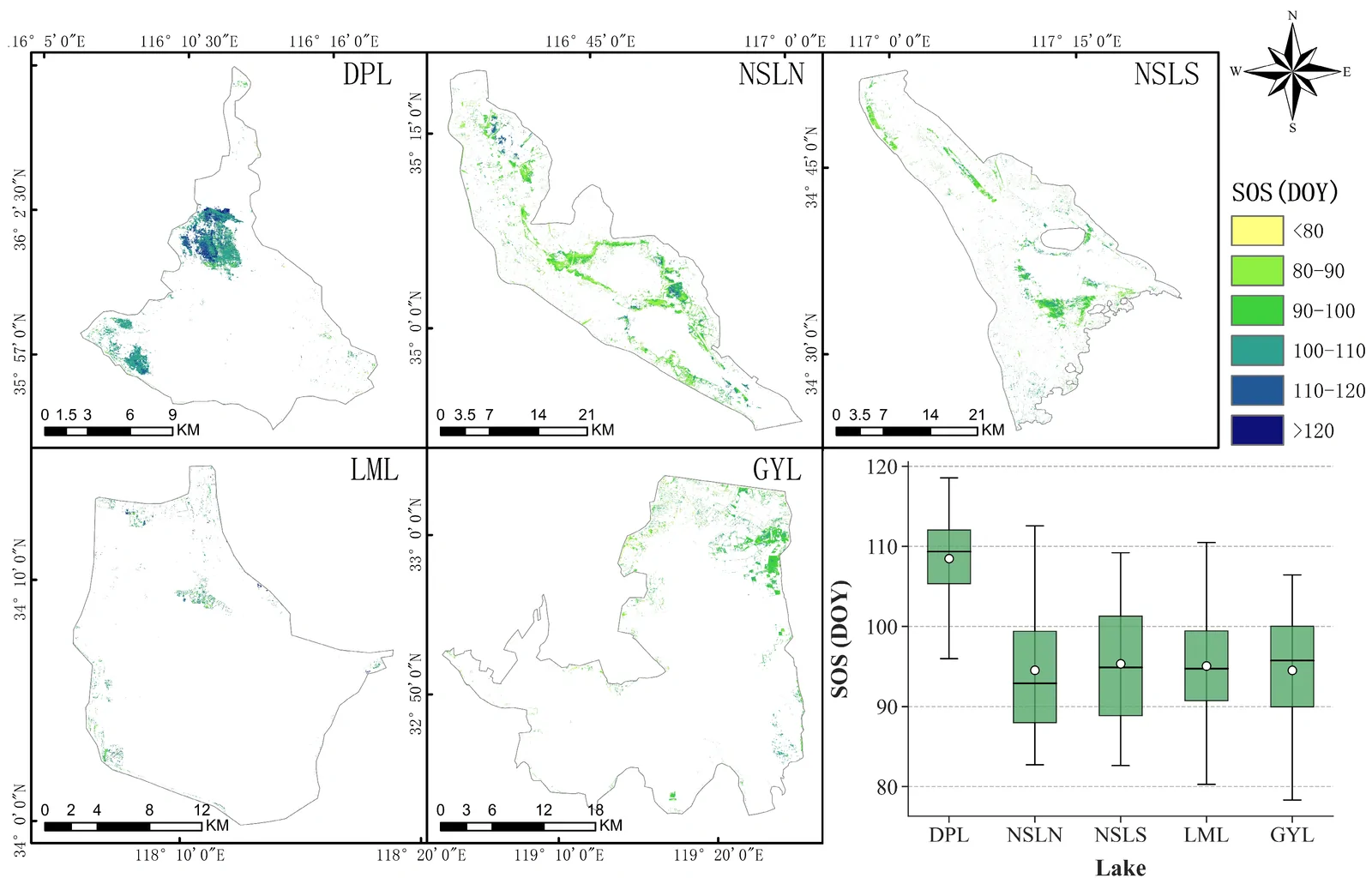
Spatial patterns of SOS for *P. crispus* in various lakes.

As shown in Figure 6, the SOS of *P. crispus* varied relatively little among the five lakes, except for the notably later onset observed in DPL. The mean SOS values of the other four lakes were highly similar, all occurring around DOY 95. DPL had the latest mean SOS, at DOY 108, whereas GYL had the earliest mean SOS, at DOY 95. Based on the unrounded values, the difference between the mean SOS values of DPL and GYL was approximately 14 days. The standard deviation of SOS within individual lakes averaged approximately 8 days, ranging from 6 days in DPL to 9 days in NSLN. These results indicate relatively small differences in mean SOS among the lakes but appreciable within-lake spatial variability, particularly in NSLN.

**Figure 7:**
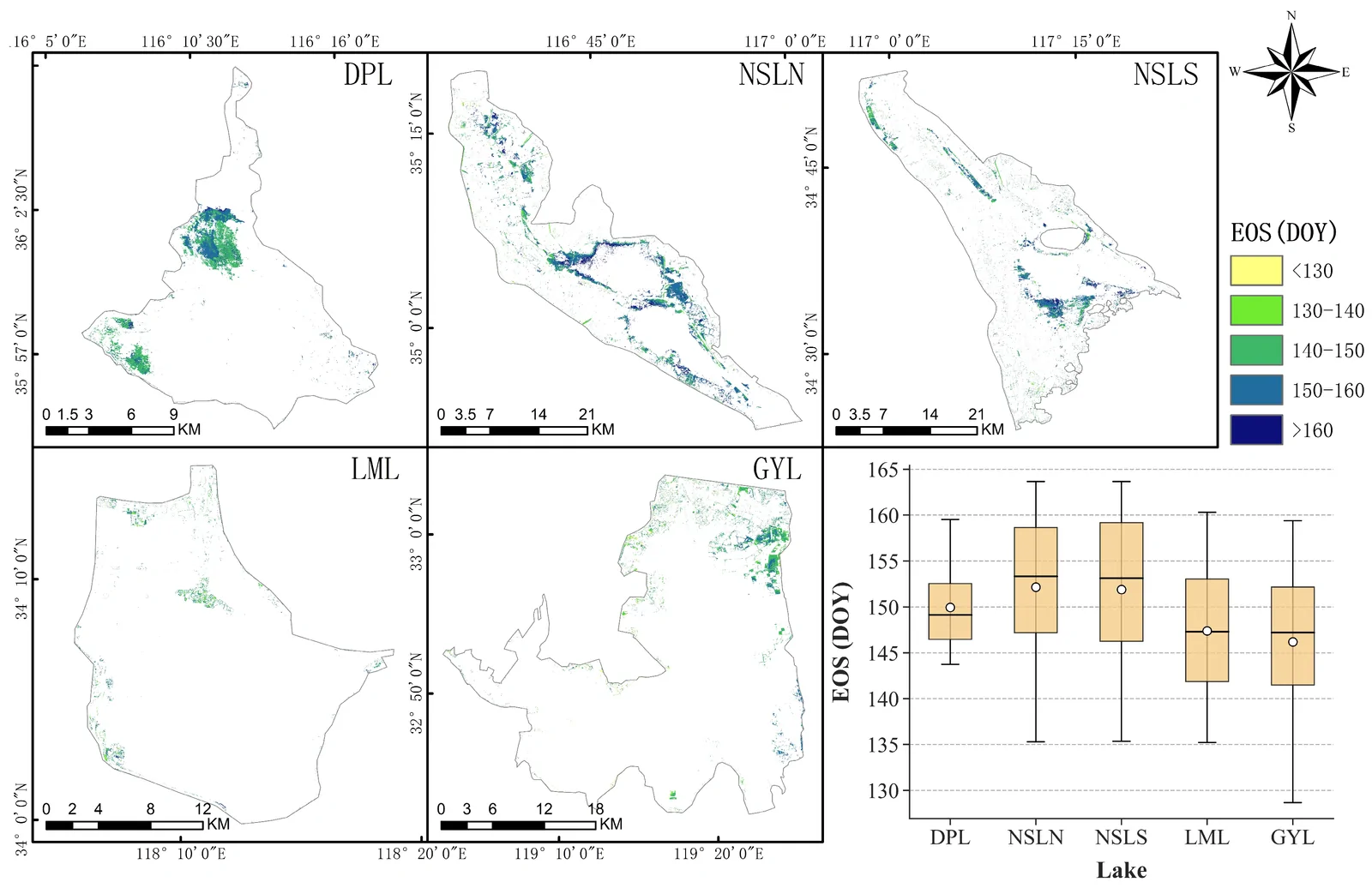
Spatial patterns of EOS for *P. crispus* in various lakes.

As shown in Figure 7, the EOS of *P. crispus* showed relatively small differences in mean timing among the five lakes, although within-lake variability was more pronounced. The difference in mean EOS among the lakes was approximately 6 days. NSLN had the latest mean EOS, at DOY 152, whereas mean EOS generally decreased southward, with GYL showing the earliest mean EOS at DOY 146. This broad spatial pattern may be associated with latitudinal variation in regional climatic conditions, although the relatively small among-lake differences suggest that other environmental factors may also contribute to local variation in phenological timing. The standard deviation of EOS within individual lakes averaged approximately 8 days, ranging from 5 days in DPL to 9 days in NSLS. Overall, the mean EOS of *P. crispus* was relatively consistent among the five lakes, despite appreciable within-lake spatial variability.

**Figure 8:**
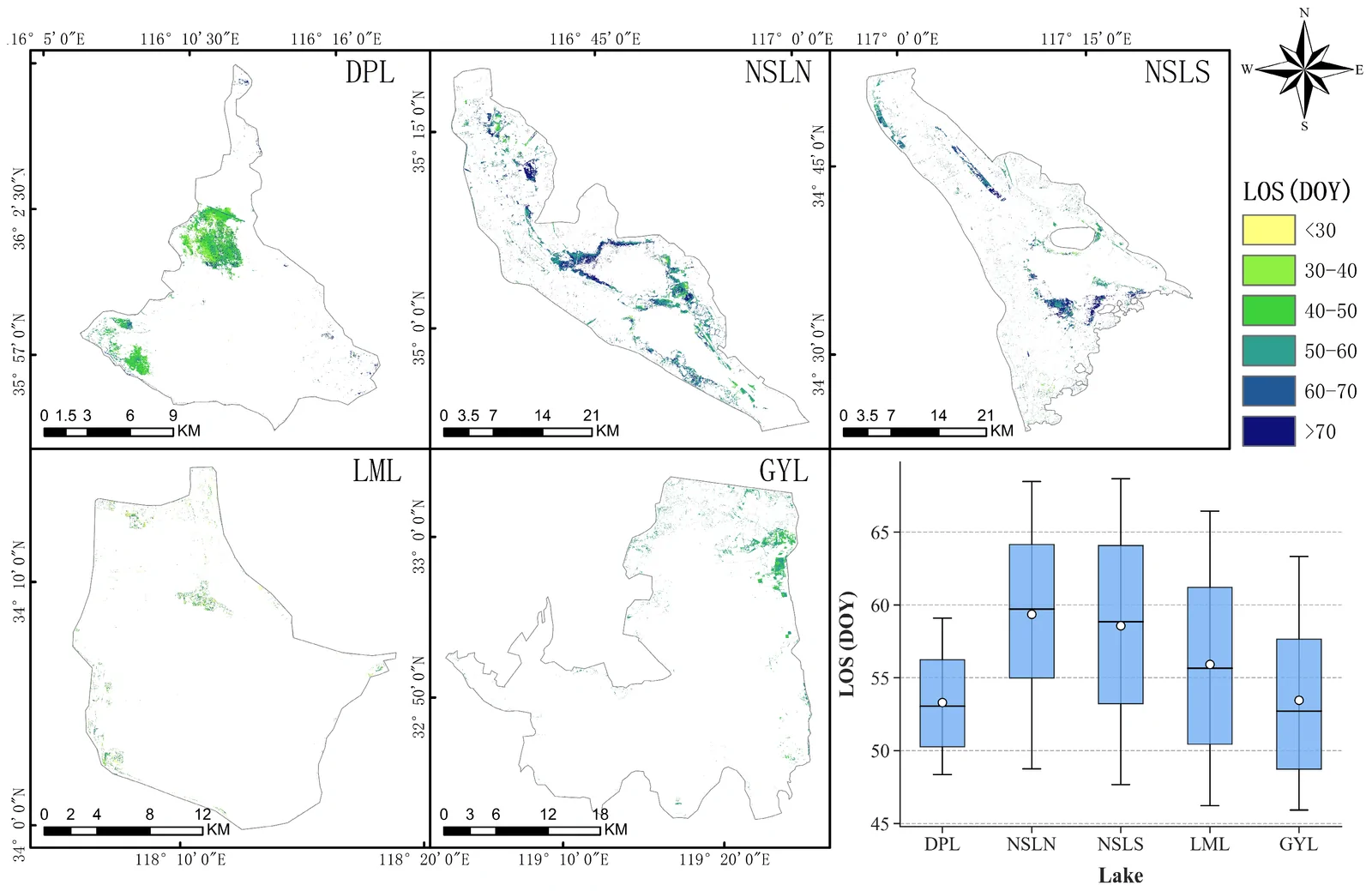
Spatial patterns of LOS for *P. crispus* in various lakes.

As shown in Figure 8, the LOS of *P. crispus* varied among the five lakes, with additional within-lake spatial heterogeneity. NSLN had the highest mean LOS, at 59 days, followed by NSLS at 59 days and LML at 56 days, whereas DPL and GYL had lower mean LOS values of 53 days. This distribution indicates relatively higher LOS at intermediate latitudes and lower values toward the northern and southern ends of the study region. The standard deviation of LOS within individual lakes averaged approximately 6 days, ranging from 3 days in DPL to 7 days in NSLS. Overall, the mean LOS of *P. crispus* showed relatively limited variation among the five lakes, although some within-lake heterogeneity was evident.

## 4. Discussion

### 4.1. Plausibility of Phenological Extraction for P. australis and P. crispus in the Lake Cluster

Because independent field-based phenological observations were unavailable, the plausibility of the retrieved phenological metrics for *P. australis* and *P. crispus* was assessed by comparing their ranges and seasonal timing with published phenological observations from the study region. For *P. australis*, SOS ranged from DOY 80 to 110, EOS from DOY 310 to 350, and LOS from approximately 220 to 250 days. Previous studies in the coastal wetlands of Yancheng, China, reported rapid growth of *P. australis* after March, gradual senescence from September, and complete withering by late November [33]. Studies in the Yancheng coastal zone similarly reported SOS around DOY 90–100 and EOS during October–November [34]. These published observations broadly overlap with the ranges retrieved in this study, providing supporting evidence for the plausibility of the extracted phenological timing of *P. australis*.

For *P. crispus*, SOS ranged from DOY 80 to 120, EOS from DOY 140 to 160, and LOS from 45 to 70 days. Previous observations in Dongping Lake (DPL) indicated vigorous growth of *P. crispus* around DOY 100–140, followed by a rapid decline during DOY 140–180 [26]. In Nansi Lake, *P. crispus* entered a rapid-growth phase and gradually emerged above the water surface from April to May, followed by senescence and decay after June [35]. The reported seasonal timing is generally consistent with the SOS and EOS ranges identified in this study, providing additional support for the plausibility of the retrieved phenological patterns of *P. crispus*.

The two species also exhibited distinct spatial patterns in phenological timing along the south–north gradient from GYL to DPL. However, these patterns did not conform to a single latitudinal response across all phenological metrics. For *P. australis*, SOS showed no significant latitudinal trend, whereas EOS occurred progressively earlier with increasing latitude, resulting in a shorter LOS toward the north. In contrast, both SOS and EOS of *P. crispus* were delayed with increasing latitude, while LOS showed no significant latitudinal gradient. Such contrasting responses suggest that latitude provides an important spatial context for phenological variation [36], but its effects may differ among species and phenological stages. Latitudinal gradients in climatic conditions and heat availability may contribute to these spatial differences, but the present study cannot directly attribute the observed patterns to aquatic thermal conditions because water-temperature observations were unavailable. Therefore, the latitudinal patterns identified here should be interpreted as spatial phenological associations rather than evidence of a uniform temperature-driven mechanism.

### 4.2. Environmental Correlates of Vegetation Phenology in the Lake Cluster

#### 4.2.1. Screening of Environmental Correlates Using Pearson Correlation

**Figure 9:**
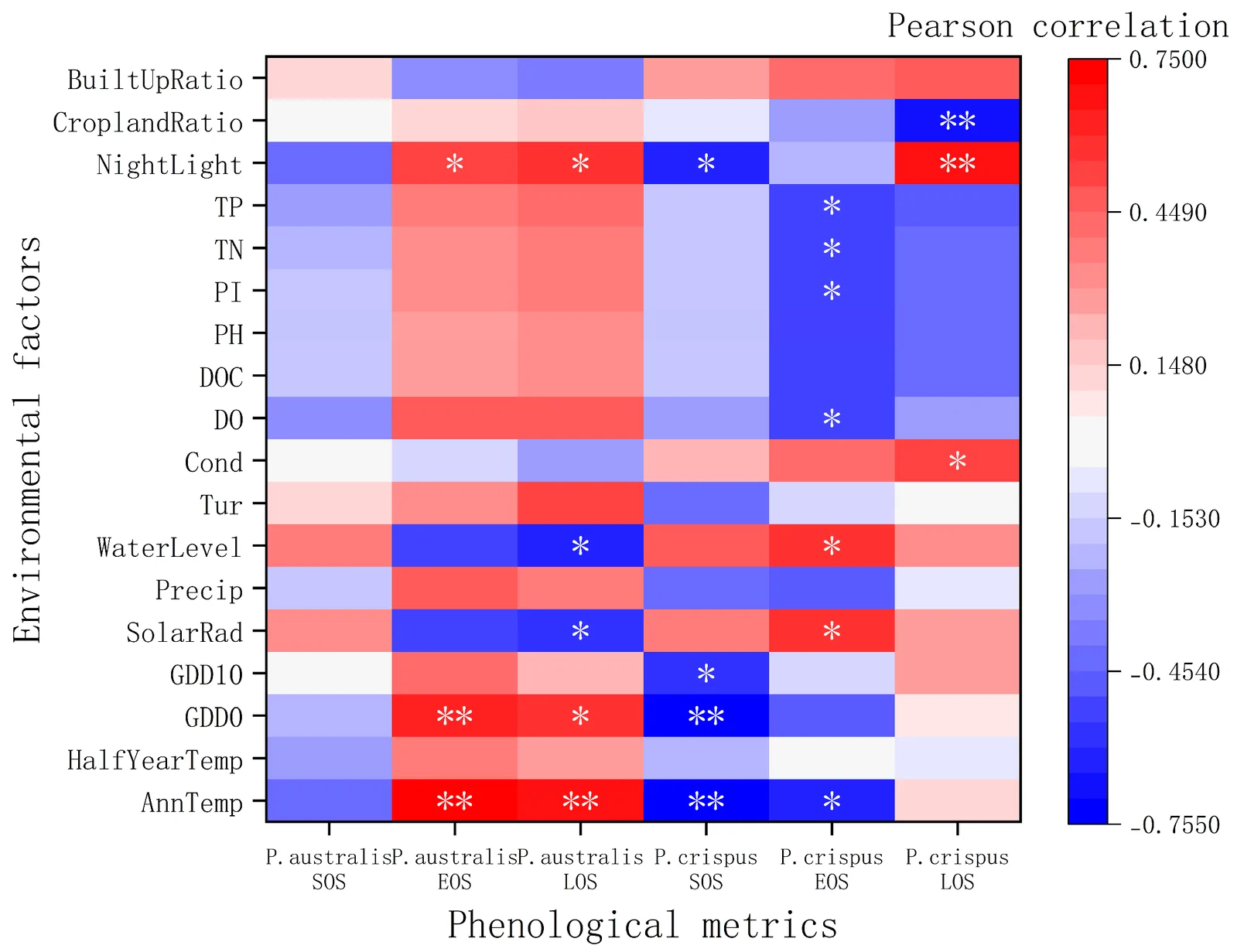
Pearson-correlation heatmap between phenological metrics and various driving factors. Note: * indicates *p* < 0.05 and ** indicates *p* < 0.01, respectively.

Pearson correlation analysis was used to examine the relationships between the phenological metrics of *P. australis* and *P. crispus* and 18 environmental factors representing meteorological, hydrological, water-environmental, and anthropogenic conditions (Figure 9). As shown in Figure 9, SOS of *P. australis* was not significantly correlated with any of the selected factors, whereas significant associations were identified for the other phenological metrics. The significant correlates also differed markedly between the two species. EOS of *P. australis* was significantly associated with mean annual temperature and nighttime light, whereas LOS was correlated with mean annual temperature, water level, and nighttime light. For *P. crispus*, SOS was significantly associated with mean annual temperature and nighttime light; EOS was correlated with mean annual temperature, water level, and total phosphorus; and LOS was primarily associated with electrical conductivity and nighttime light.

Overall, meteorological factors showed significant associations with aquatic vegetation phenology, with *P. australis* and *P. crispus* showing distinct response patterns. Mean annual temperature was significantly positively correlated with EOS and LOS of *P. australis*, indicating that higher mean annual air temperature was associated with later senescence and a longer growing season. In contrast, mean annual temperature was significantly negatively correlated with SOS and EOS of *P. crispus*, indicating that warmer conditions were associated with earlier onset and senescence. Hydrological and water-environmental factors were also associated with phenological variation: water level and total phosphorus were associated with EOS of *P. crispus*, whereas water level was negatively associated with LOS of *P. australis*. Electrical conductivity was associated only with LOS of *P. crispus*. As a proxy for anthropogenic disturbance, nighttime light was significantly correlated with multiple phenological metrics, indicating associations with phenological timing across multiple developmental stages.

#### 4.2.2. Relative Importance of Environmental Predictors Based on PLSR

**Figure 10:**
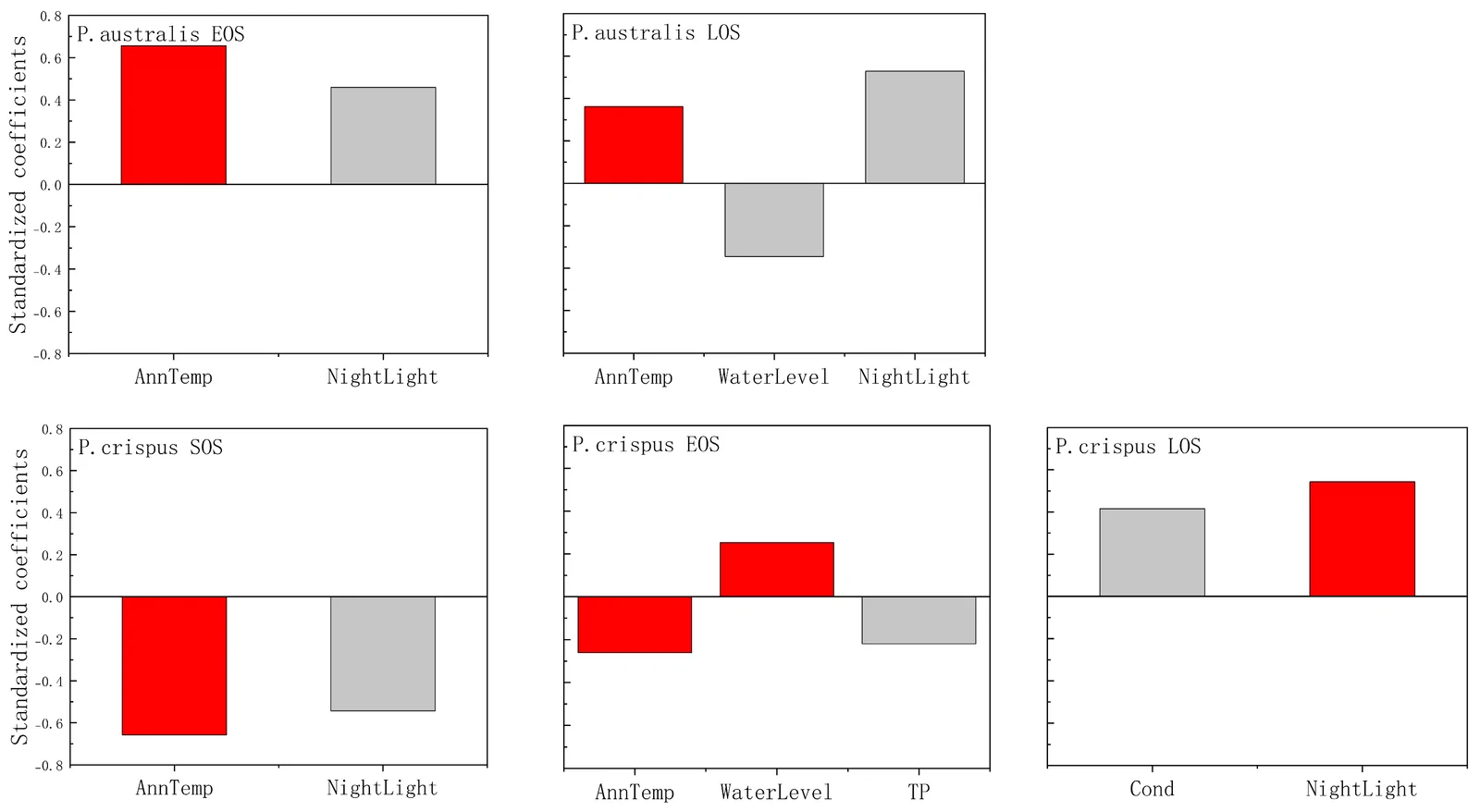
Standardized PLSR coefficients and VIP values for the phenological metrics of *P. australis* and *P. crispus.* Note: represents relatively important predictors; red bars indicate VIP values > 1; grey bars indicate VIP values ≤ 1.

PLSR was performed using the predictors selected through Pearson correlation analysis. As shown in Figure 10, the direction and relative importance of the predictors varied substantially among phenological metrics. Mean annual temperature was positively associated with EOS and LOS of *P. australis* but negatively associated with SOS and EOS of *P. crispus*. Water level was negatively associated with LOS of *P. australis* and positively associated with EOS of *P. crispus*, whereas total phosphorus and electrical conductivity were associated only with the phenological metrics of *P. crispus*. Nighttime light was associated with delayed phenological timing and longer LOS in *P. australis*, but with earlier SOS and longer LOS in *P. crispus*, indicating contrasting associations between the two species.

Based on VIP values, mean annual temperature showed the highest overall importance among the linear predictors, whereas water level, total phosphorus, electrical conductivity, and nighttime light showed comparatively lower importance. Overall, the PLSR results indicated that the relative importance and direction of environmental associations varied among species and phenological stages, with mean annual temperature showing the most consistent importance across the analyzed metrics.

#### 4.2.3. Nonlinear Associations Based on the XGBoost-SHAP Model

We evaluated the cross-lake generalizability of XGBoost models via leave-one-lake-out cross-validation, and the out-of-fold performance metrics for each phenological model are summarized in Table S4 (Supporting Materials). For *P. australis*, all three phenological models (SOS, EOS, LOS) produced negative out-of-fold R^2^_cv_, indicating very limited predictive power when extrapolating to unseen lakes. For *P. crispus*, the out-of-fold R^2^_cv_ was −0.23 for SOS, 0.06 for EOS, and 0.29 for LOS; only the LOS model achieved weak predictive performance. Collectively, these metrics demonstrate that the XGBoost models had limited capacity for cross-lake prediction, which contextualizes the subsequent SHAP interpretation.

**Figure 11:**
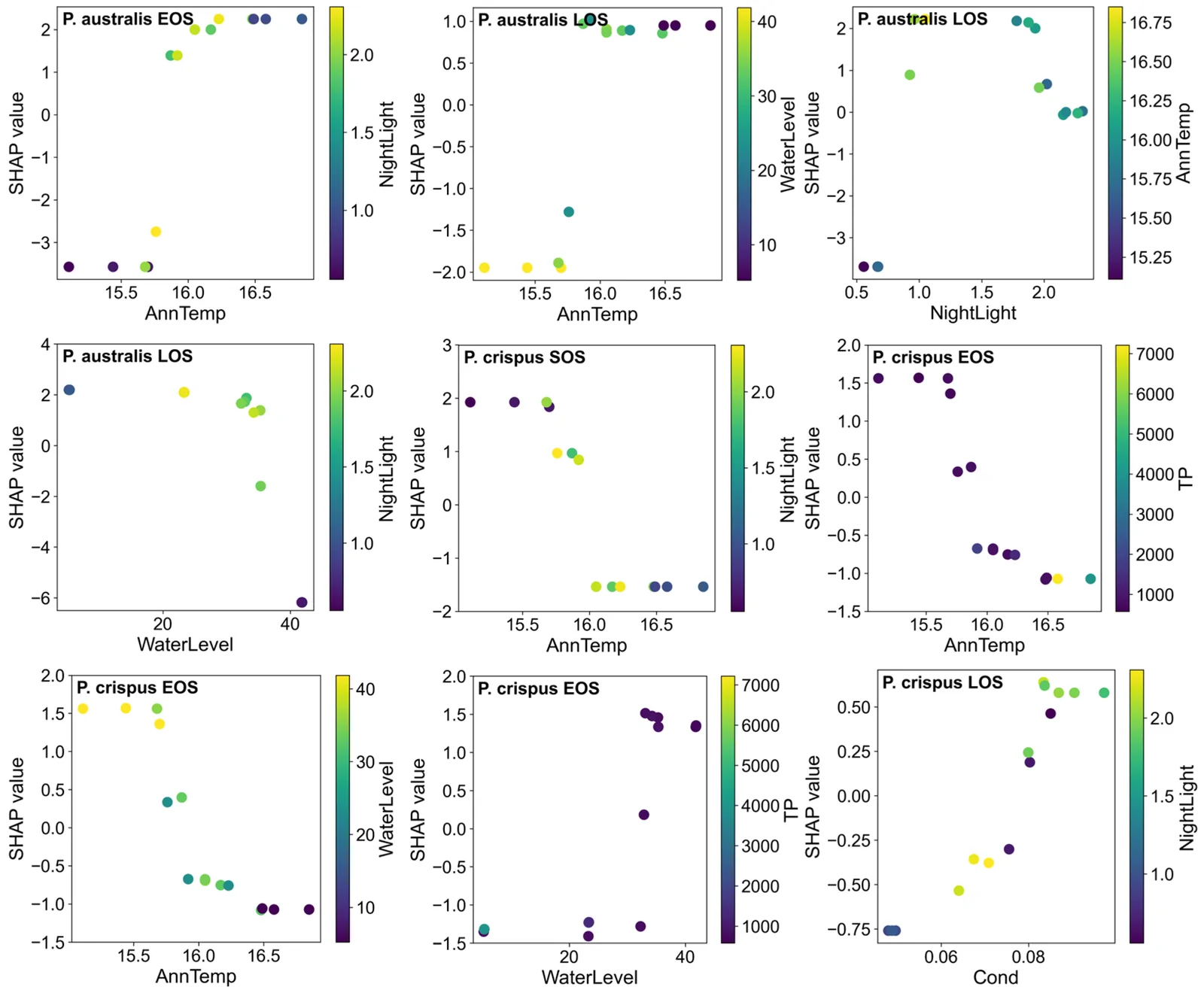
SHAP dependence plots illustrating potential nonlinear marginal associations between selected environmental predictors and the phenological metrics of *P. australis* and *P. crispus.* This exploratory SHAP analysis should be interpreted with caution owing to the limited lake-year sample size.

The SHAP dependence plots showed distinct nonlinear marginal associations between the selected environmental predictors and phenological metrics (Figure 11). This SHAP analysis is exploratory and should be interpreted cautiously given the limited lake-year sample size. For *P. australis*, annual mean temperature showed pronounced nonlinear associations with both EOS and LOS. For EOS, SHAP contributions shifted from predominantly negative to positive values with increasing temperature, showing an apparent shift near 16 °C from the SHAP model outputs; this pattern is not interpreted as an ecological threshold and requires verification with larger datasets. For LOS, the SHAP contribution of annual mean temperature similarly shifted from negative to positive values, while water level and nighttime light also showed nonlinear marginal associations.

For *P. crispus*, annual mean temperature showed predominantly negative nonlinear associations with both SOS and EOS, with SHAP contributions generally decreasing as temperature increased. EOS also showed positive nonlinear associations with water level and total phosphorus, whereas LOS exhibited a relatively weak positive association with electrical conductivity that tended to approach saturation. Nighttime light also showed a nonlinear association with LOS.

Overall, the SHAP dependence plots indicated that the associations between environmental predictors and phenological metrics were not uniformly linear, and that their direction and magnitude varied among species and phenological stages. Given the limited sample size and the limited cross-lake predictive performance of the XGBoost model, these nonlinear patterns should be interpreted as exploratory model-derived associations rather than robust ecological thresholds or causal effects.

### 4.3. Differential Latitudinal Responses of P. australis and P. crispus

**Figure 12:**
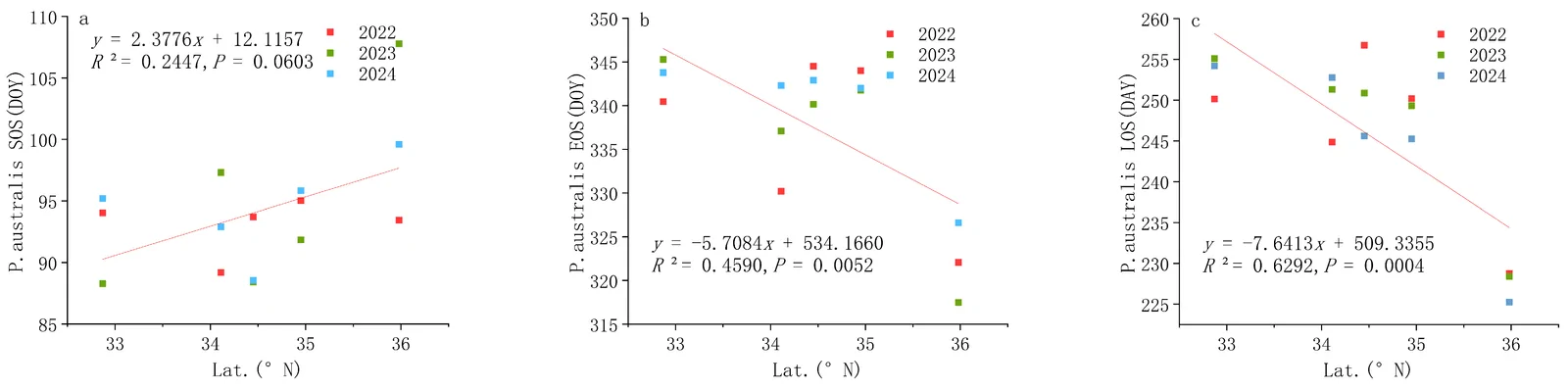

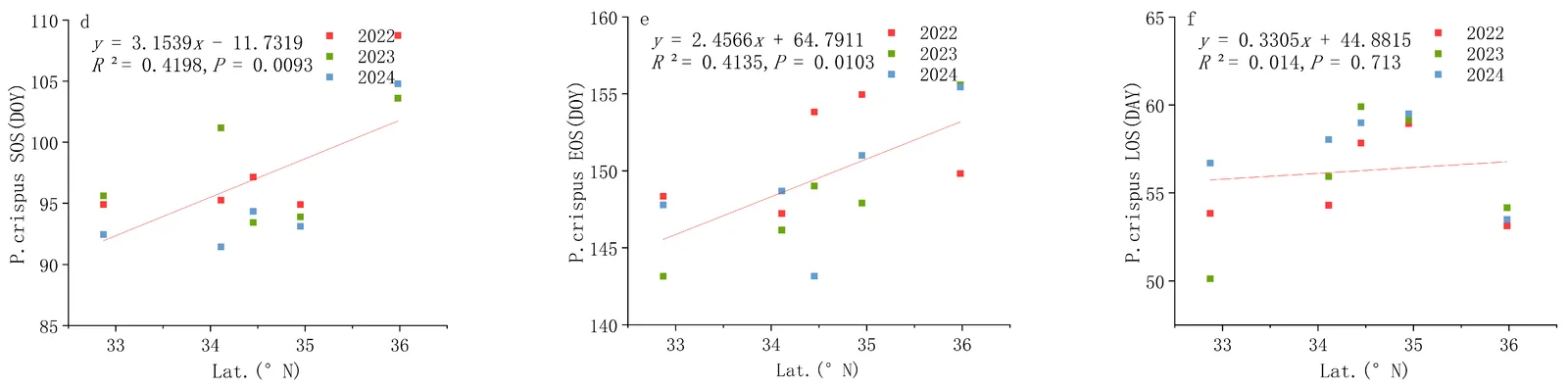
Linear regression fitting results between latitude and SOS (**a**), EOS (**b**), LOS (**c**) of *P. australis*, as well as SOS (**d**), EOS (**e**), LOS (**f**) of *P. crispus.* Points are colour-coded by year to distinguish repeated lake-year samples. The red solid lines denote linear regression lines.

Based on linear regressions between latitude and SOS, EOS, and LOS of *P. australis* (Figure 12a–c), the latitudinal patterns of these phenological metrics were quantitatively assessed. The SOS–latitude relationship showed a positive slope, with SOS delayed by an average of 2.38 days per 1° increase in latitude, indicating a tendency for SOS to occur later at higher latitudes. However, the marginal significance (*p* = 0.0603) suggests only a weak latitudinal dependence of SOS. This non-significant relationship suggests that the latitudinal gradient alone did not explain the observed variation in spring phenology [37]. In contrast, EOS showed a significant negative relationship with latitude, advancing by an average of 5.71 days per 1° increase in latitude (*R*^2^ = 0.4590, *p* < 0.01), indicating a pronounced latitudinal pattern in autumn senescence. LOS also decreased significantly with latitude, shortening by 7.64 days per 1° increase (*R*^2^ = 0.6292, *p* < 0.001), representing the strongest latitudinal relationship among the three phenological metrics. Overall, except for SOS, *P. australis* phenology exhibited a pronounced latitudinal pattern, with shorter growing seasons in northern lakes and longer growing seasons in southern lakes. The latitudinal variation in LOS was closely associated with the latitudinal variation in EOS, suggesting that autumn phenology contributed more strongly than spring phenology to the latitudinal variation in growing-season duration.

To evaluate the influence of repeated annual observations within individual lakes, we performed a sensitivity analysis using three-year averaged phenological values for each lake (*n* = 5). The sign of regression slopes for all phenological metrics remained consistent with the lake-year (*n* = 15) model, demonstrating the robustness of these core latitudinal patterns. Differences in *R*^2^ and *p*-values between the two datasets were expected and attributable to the reduced sample size of lake-averaged data. Detailed regression statistics for both datasets are provided in Table S2 in Supplementary Materials.

Based on linear regressions between latitude and SOS, EOS, and LOS of *P. crispus* (Figure 12d–f), the latitudinal patterns and statistical significance of these phenological metrics were quantitatively assessed. SOS of *P. crispus* showed a significant positive relationship with latitude, with SOS delayed by an average of 3.15 days per 1° increase in latitude (*R*^2^ = 0.4198, *p* = 0.0093). EOS also increased significantly with latitude, being delayed by 2.46 days per 1° increase (*R*^2^ = 0.4135, *p* = 0.0103). The similar *R*^2^ and *p* values for SOS and EOS indicate that latitude explained comparable proportions of their variation. This contrasting latitudinal pattern is broadly consistent with the temperature sensitivity of *P. crispus* senescence reported in previous studies, although the underlying thermal mechanism could not be directly evaluated in this study. Notably, the LOS–latitude slope was close to zero and non-significant (*p* > 0.05), indicating no significant latitudinal gradient in LOS of *P. crispus*. This may occur because latitudinal variation in environmental conditions shifts the timing of both growth onset and senescence without substantially altering the overall duration of the growing season [38].

Comparison of the two species revealed distinct latitudinal responses in phenology. During the spring green-up period, SOS of *P. australis* showed a weak latitudinal response, whereas SOS of *P. crispus* was more strongly associated with latitude, indicating a stronger statistical association between spring phenology and latitude for the submerged macrophyte *P. crispus*. During senescence, the two species exhibited opposite EOS responses: EOS of *P. australis* advanced significantly with increasing latitude, whereas EOS of *P. crispus* was significantly delayed, highlighting their contrasting responses to the latitudinal climatic gradient. For LOS, *P. australis* showed a highly significant decrease with increasing latitude, indicating a pronounced latitudinal gradient, whereas *P. crispus* showed no significant latitudinal variation in LOS. These contrasting patterns indicate that the two macrophytes exhibit different latitudinal responses in growing-season duration.

Latitudinal gradients are accompanied by differences in regional air temperature, solar radiation, and may also coincide with differences in aquatic thermal conditions. The contrasting phenological responses may partly reflect physiological differences between the emergent macrophyte *P. australis* and the cool-water submerged macrophyte *P. crispus*. As a cool-water submerged species, *P. crispus* exhibits an early-season growth strategy, and previous studies have reported that spring temperature can influence turion germination and the onset of spring growth [39]. This documented temperature sensitivity may partly explain the latitudinal pattern in SOS observed here. Meanwhile, *P. crispus* has limited tolerance to high temperatures, and previous studies have reported that water temperatures above approximately 25 °C can accelerate senescence and increase mortality [40]. This previously reported thermal sensitivity may also be consistent with the observed latitudinal variation in EOS, with later EOS at higher latitudes and earlier EOS at lower latitudes. The concurrent delays in SOS and EOS with increasing latitude largely offset each other in LOS, resulting in no significant latitudinal gradient in LOS.

In contrast, *P. australis* is an emergent rhizomatous plant that overwinters through underground rhizomes. Its spring emergence is influenced by temperature as well as local environmental conditions, including riparian soil moisture and water levels. Local hydrological variability may therefore weaken the influence of the latitudinal climatic gradient on SOS, resulting in a non-significant SOS–latitude relationship [41]. In autumn, the earlier decline in regional thermal conditions at higher latitudes may reduce the conditions favorable for sustained photosynthetic activity in *P. australis*, contributing to earlier EOS. Consequently, latitudinal variation in EOS was closely associated with the latitudinal pattern of LOS, producing longer growing seasons in the south and shorter growing seasons in the north [42]. Overall, the latitudinal pattern of *P. australis* phenology was dominated by EOS, with significant latitudinal variation in EOS and LOS but not SOS. In contrast, *P. crispus* showed concurrent latitudinal shifts in SOS and EOS, which largely offset each other in LOS, resulting in no significant latitudinal gradient in growing-season duration. Because water-temperature observations were unavailable in this study, these contrasting patterns should be interpreted as latitudinal phenological associations rather than direct evidence of water-temperature-driven mechanisms.

### 4.4. Limitations

This study focused on divergent latitudinal responses of phenology between emergent and submerged macrophytes, based on data from five representative lakes spanning a latitudinal gradient across the North China Plain lake region during 2022–2024. However, the analysis did not encompass all lakes in the region or a longer time series, limiting our ability to characterize temporal variability. Moreover, the 15 lake-year samples are derived from only five lakes; repeated annual measurements within individual lakes may be statistically correlated, which limits spatial representativeness and extrapolation of latitudinal patterns beyond the study area. In addition, cloud contamination and mixed-pixel effects associated with landscape fragmentation inevitably introduced uncertainty into the mapping of *P. australis* and *P. crispus* from Sentinel-2 imagery, which could subsequently affect phenological retrieval. Although missing observations were gap-filled before fitting the NDVI time series with the DL function, residual data gaps may have affected the fitted NDVI trajectories and introduced uncertainty into the derived phenological metrics. The extracted SOS, EOS, and LOS have not been fully validated against independent field phenological observations, so the accuracy of these remotely sensed phenological metrics remains to be further assessed. Moreover, water-temperature observations were unavailable, and air temperature was therefore used as a climatic indicator. Consequently, the observed temperature–phenology associations cannot be directly interpreted as aquatic thermal responses. The limited sample size (15 lake-year observations) also constrains the robustness and generalizability of the XGBoost-SHAP results, which should therefore be interpreted as exploratory nonlinear associations. Future studies could improve the robustness of these findings by expanding the study area, incorporating remote-sensing datasets with higher spatiotemporal resolution, and integrating more extensive in situ phenological observations.

## 5. Conclusions

*P. australis* and *P. crispus* exhibited distinct spatial distributions and contrasting latitudinal phenological responses across the shallow lakes of the North China Plain. *P. australis* showed earlier EOS and shorter LOS toward higher latitudes, whereas both SOS and EOS of *P. crispus* were delayed with increasing latitude, resulting in no significant latitudinal gradient in LOS.

Environmental associations were species- and stage-specific. Mean annual temperature showed the highest overall importance among the linear predictors, while XGBoost-SHAP revealed nonlinear marginal associations that varied among phenological stages, including an apparent change in the modeled EOS response of *P. australis* near 16 °C. This feature is an exploratory, model-derived result and should not be interpreted as a confirmed biological threshold.

These findings highlight contrasting phenological responses of emergent and submerged macrophytes along the latitudinal gradient and provide a basis for phenology-informed ecological monitoring and management of shallow lakes in the North China Plain.

## Figures and Tables

**Table 1:** Data sources used in this study.

	Data	Source	Application
Sentinel-2 L2A		Google Earth Engine (GEE, Google Inc., Mountain View, CA, USA) platform	Vegetation classification, phenological metric extraction
Lake vector boundary		HydroSHEDS platform [https://www.hydrosheds.org/products/hydrolakes#downloads (accessed on 15 Mar 2026)]	Lake boundary extraction
Auxiliary data	Meteorological data	National Cryosphere Desert Data Center [https://www.ncdc.ac.cn/portal/metadata/21691d03-bef2-4800-924e-5614e7268b87 (accessed on 15 Mar 2026)]	Environmental association analysis
	Solar radiation data	National Tibetan Plateau Data Center [https://doi.org/10.11888/Meteoro.tpdc.270961 (accessed on 15 Mar 2026)]	
	Accumulated temperature data	ERA5-Land daily aggregated dataset (ECMWF/ERA5_LAND/DAILY_AGGR)	
	Water quality data	Scientific Data platform [https://doi.org/10.6084/m9.figshare.27626286.v2 (accessed on 15 Mar 2026)]	
	Water level data	Science Data Bank platform [https://www.scidb.cn/en/detail?dataSetId=b102eb5d632c4268a6030492b822127f (accessed on 15 Mar 2026)]	
	Nighttime light data	National Tibetan Plateau Data Center [https://data.tpdc.ac.cn/zh-hans/data/a0a268e2-3c6a-42cb-9b15-7b81d5e9a833 (accessed on 15 Mar 2026)]	
	Land-use data	China’s annual 30-m land-cover dataset released by Wuhan University [https://zenodo.org/records/12779975 (accessed on 15 Mar 2026)]	

**Table 2:** Remote sensing images used for vegetation classification.

Lake	Image Date (*P. australis* Extraction)	Image Date (*P. crispus* Extraction)
DPL	8 July 2022; 18 July 2023; 6 August 2024	4 May 2022; 13 June 2022; 29 April 2023; 13 June 2023; 13 May 2024; 17 June 2024
NSLN, NSLS	7 August 2022; 7 August 2023; 11 August 2024	4 May 2022; 18 June 2022; 14 May 2023; 13 June 2023; 13 May 2024; 7 June 2024
LML	4 August 2022; 5 July 2023; 29 July 2024	16 May 2022; 20 June 2022; 21 May 2023; 15 June 2023; 10 May 2024; 9 June 2024
GYL	4 August 2022; 5 July 2023; 29 July 2024	6 May 2022; 15 June 2022; 1 May 2023; 15 June 2023; 10 May 2024; 9 June 2024

**Table 3:** Remote sensing indices and their calculation formulas.

Remote-Sensing Index	Calculation Formula
NDVI	(NIR − Red)/(NIR + Red)
EVI	2.5 × (NIR − Red)/(NIR + 6 × Red − 7.5 × Blue + 1)
NDWI	(Green − NIR)/(Green + NIR)
NDAVI	(NIR − Green)/(NIR + Green)

**Table 4:** Area of *P. australis* and *P. crispus* in the lakes and their three-year average.

Lake	Year	*P. australis*				*P. crispus*			
		Area /km^2^	Proportion	Mean Area/km^2^	Mean Proportion	Area /km^2^	Proportion	Mean Area /km^2^	Mean Proportion
DPL	2022	7.85	5.06%	5.5	3.55%	17.66	11.39%	6.24	4.03%
	2023	3.2	2.07%			0.3	0.20%		
	2024	5.44	3.51%			0.77	0.49%		
NSLN	2022	40.26	6.48%	33.87	5.45%	60.24	9.69%	45.76	7.36%
	2023	23.27	3.74%			35.53	5.72%		
	2024	38.07	6.13%			41.49	6.68%		
NSLS	2022	16.39	2.51%	21.18	3.24%	39.66	6.06%	35.34	5.40%
	2023	15.83	2.42%			48.08	7.35%		
	2024	31.33	4.79%			18.26	2.79%		
LML	2022	3.94	1.28%	4.22	1.37%	3.32	1.08%	2.15	0.70%
	2023	4.5	1.46%			1.19	0.39%		
	2024	4.2	1.36%			1.93	0.63%		
GYL	2022	11.7	1.59%	14.35	1.95%	15.77	2.15%	13.64	1.86%
	2023	14.87	2.02%			15.73	2.14%		
	2024	16.49	2.25%			9.41	1.28%		

**Table 5:** Extraction accuracy of *P. australis* and *P. crispus*.

Lake	Year	*P. australis*		*P. crispus*
		OA	Kappa	PA
DPL	2022	94.31%	0.80	98.64%
	2023	98.51%	0.90	95.22%
	2024	99.84%	0.99	84.91%
NSLN	2022	99.22%	0.98	96.67%
	2023	99.08%	0.97	94.22%
	2024	99.32%	0.98	86.25%
NSLS	2022	97.98%	0.96	94.29%
	2023	99.20%	0.98	98.70%
	2024	96.31%	0.92	91.62%
LML	2022	99.44%	0.94	89.88%
	2023	99.74%	0.95	92.16%
	2024	99.45%	0.96	86.72%
GYL	2022	99.92%	0.99	96.75%
	2023	97.91%	0.96	97.21%
	2024	98.92%	0.98	97.49%

## Data Availability

The Sentinel-2 Level-2A imagery used in this study is openly available through the Google Earth Engine (GEE) platform. The intermediate processed datasets generated during this study are available from the corresponding author upon reasonable request due to their large data volume.

## Supplementary Materials

The following supporting information can be downloaded at https://www.mdpi.com/article/10.3390/plants15203240/s1: Table S1. Producer accuracy and user accuracy for *Phragmites australis* and *Potamogeton crispus* mapping. Table S2. Linear regression statistics between latitude and phenological metrics for two aquatic plant species, based on lake-year observations (*n* = 15) and three-year lake-averaged values (*n* = 5). Table S3. Cumulative explained variance (*R*^2^Xcum and *R*^2^Ycum) derived from PLSR models for each phenological metric. Table S4. Leave-one-lake-out cross-validation performance metrics of XGBoost models for each phenological metric.
