# Supplementary material for: Latitudinal Variation in the Phenology of the Emergent Species Phragmites australis and the Submerged Species Potamogeton crispus in Shallow Lakes of the North China Plain
Source: Plants (Basel). 2026 Oct 21;15(20):3240. doi: 10.3390/plants15203240 (PMC13709194; doi:10.3390/plants15203240)
Supplement: Supplementary file 1 [file plants-15-03240-s001.zip › plants-4602502-supplementary.pdf]

Table S1. Producer accuracy and user accuracy for *Phragmites australis* and *Potamogeton crispus* mapping.

Lake	Year	*P. australis*		*P. crispus*
		PA	UA	UA
DPL	2022	93.07%	98.07%	93.50%
	2023	95.56%	92.63%	88.50%
	2024	99.62%	99.87%	92.50%
NSLN	2022	96.91%	92.43%	93.00%
	2023	95.56%	92.63%	93.50%
	2024	89.25%	90.36%	90.00%
NSLS	2022	98.88%	98.71%	96.00%
	2023	92.17%	97.14%	94.00%
	2024	93.62%	90.29%	90.00%
LML	2022	89.18%	88.35%	96.00%
	2023	91.24%	93.68%	92.00%
	2024	90.32%	89.48%	92.50%
GYL	2022	94.32%	95.13%	94.50%
	2023	94.43%	95.46%	93.00%
	2024	95.76%	96.31%	94.50%

Table S2. Linear regression statistics between latitude and phenological metrics for two aquatic plant species, based on lake-year observations (n = 15) and three-year lake-averaged values (n = 5).

Metric		Slope (n=15)	R^2 (n=15)	P (n=15)	Slope (n=5)	R^2 (n=5)	P (n=5)
P. australis	SOS	2.38	0.24	0.06	2.38	0.52	0.17
	EOS	-5.71	0.46	<0.01	-5.71	0.53	0.17
	LOS	-7.64	0.63	<0.001	-7.64	0.69	0.08
P. crispus	SOS	3.15	0.42	<0.01	3.15	0.53	0.16
	EOS	2.46	0.41	0.01	2.46	0.90	0.01
	LOS	0.33	0.01	0.71	0.33	0.02	0.83

Table S3. Cumulative explained variance (R^2X_{cum} and R^2Y_{cum}) derived from PLSR models for each phenological metric.

Metric		Optimal latent components	R^2 X_{cum}	R^2 Y_{cum}
P. australis	EOS	1	56.97	72.92
	LOS	1	89.61	79.83
P. crispus	SOS	1	57.21	83.00
	EOS	2	80.80	40.80
	LOS	1	64.10	59.80

Table S4. Leave-one-lake-out cross-validation performance metrics of XGBoost models for each phenological metric.

Metric		R^2_{cv}	RMSE	MAE
P. australis	EOS	-0.36	10.02	7.23
	LOS	-0.42	11.70	8.51
P. crispus	SOS	-0.23	5.51	4.10
	EOS	0.06	3.79	3.14
	LOS	0.29	2.41	2.14
